# Silencing of *Syntaxin 1A* in the Dopaminergic Neurons Decreases the Activity of the Dopamine Transporter and Prevents Amphetamine-Induced Behaviors in *C. elegans*

**DOI:** 10.3389/fphys.2018.00576

**Published:** 2018-05-22

**Authors:** Ambra Lanzo, Bryan D. Safratowich, Sirisha R. Kudumala, Ivan Gallotta, Giuseppina Zampi, Elia Di Schiavi, Lucia Carvelli

**Affiliations:** ^1^Institute of Biosciences and Bioresources, National Research Council (CNR), Naples, Italy; ^2^Department of Biomedical Sciences, University of North Dakota, Grand Forks, ND, United States; ^3^Brain Institute, Florida Atlantic University, Jupiter, FL, United States; ^4^Institute of Genetics and Biophysics, National Research Council (CNR), Naples, Italy; ^5^Harriet L. Wilkes Honors College, Florida Atlantic University, Jupiter, FL, United States

**Keywords:** Syntaxin-1A, dopamine, amphetamine, *C. elegans*, dopamine transporter

## Abstract

The dopamine transporter (DAT) is a cell membrane protein whose main function is to reuptake the dopamine (DA) released in the synaptic cleft back into the dopaminergic neurons. Previous studies suggested that the activity of DAT is regulated by allosteric proteins such as Syntaxin-1A and is altered by drugs of abuse such as amphetamine (Amph). Because *Caenorhabditis elegans* expresses both DAT (DAT-1) and Syntaxin-1A (UNC-64), we used this model system to investigate the functional and behavioral effects caused by lack of expression of *unc-64* in cultured dopaminergic neurons and in living animals. Using an inheritable RNA silencing technique, we were able to knockdown *unc-64* specifically in the dopaminergic neurons. This cell-specific knockdown approach avoids the pleiotropic phenotypes caused by knockout mutations of *unc-64* and ensures the transmission of dopaminergic specific *unc-64* silencing to the progeny. We found that, similarly to *dat-1* knockouts and *dat-1* silenced lines, animals with reduced *unc-64* expression in the dopaminergic neurons did not respond to Amph treatment when tested for locomotor behaviors. Our *in vitro* data demonstrated that in neuronal cultures derived from animals silenced for *unc-64*, the DA uptake was reduced by 30% when compared to controls, and this reduction was similar to that measured in neurons isolated from animals silenced for *dat-1* (40%). Moreover, reduced expression of *unc-64* in the dopaminergic neurons significantly reduced the DA release elicited by Amph. Because in *C. elegans* DAT-1 is the only protein capable to reuptake DA, these data show that reduced expression of *unc-64* in the dopaminergic neurons decreases the capability of DAT in re-accumulating synaptic DA. Moreover, these results demonstrate that decreased expression of *unc-64* in the dopaminergic neurons abrogates the locomotor behavior induced by Amph. Taken together these data suggest that Syntaxin-1A plays an important role in both functional and behavioral effects caused by Amph.

## Introduction

The DAT is a plasma membrane protein which reuptakes the DA released in the synaptic cleft back into the neurons. By so doing, DAT plays a central role in controlling the extracellular content of DA and regulating the amplitude of the dopaminergic signaling. DAT has been involved in the etiology and treatment of various neurologic disorders including schizophrenia, attention deficit hyperactivity disorder (ADHD) and Parkinson’s disease (Miller et al., 1999; Wang et al., 2013; Markota et al., 2014). Interestingly, DAT is also one of the major targets for psychostimulants such as cocaine and Amph; and while cocaine is a DAT blocker, Amph is also a DAT substrate, thus preventing DA uptake, and a releaser causing DA release by inducing reverse transport of DA from inside to outside the neurons through DAT (Khoshbouei et al., 2003). The resulting increase of extracellular DA is believed to be the first step that ultimately generates the behavioral outcomes produced by Amph.

Previous data showed that the regulatory effects of Amph on DAT require the participation of ancillary proteins such as Syntaxin-1A (Binda et al., 2008). Syntaxin-1A is a member of the SNARE (soluble *N*-ethylmaleimide-sensitive factor attachment protein receptor) proteins complex involved in the process of membrane vesicle fusion which leads to exocytosis and thus neurotransmitter release (Salaün et al., 2004). The neuronal specific isoform can bind to and regulate different plasma membrane proteins including ion channels (Naren et al., 1998; Arien et al., 2003; Condliffe et al., 2004; Tsuk et al., 2004) and neurotransmitter transporters (Deken et al., 2000; Geerlings et al., 2001; Haase et al., 2001; Horton and Quick, 2001; Quick, 2003, 2006; Sung et al., 2003; Wang et al., 2003; Fan et al., 2006). Studies focused on the DAT/ Syntaxin-1A interaction have showed that Syntaxin-1A directly binds at the N-terminal domain of DAT (Lee et al., 2004), reduces the capability of DAT to reuptake DA (Cervinski et al., 2010) and increases the ability of Amph to cause DA efflux (Binda et al., 2008). However, few studies have been performed to assess whether the interaction and/or lack of interaction between Syntaxin-1A and DAT may cause behavioral outcomes in living animals (Carvelli et al., 2008; Cartier et al., 2015).

Previously, we showed that, like in mammals, the *C. elegans* Syntaxin-1A homolog UNC-64 interacts with the *C. elegans* DAT (DAT-1) and regulates the electrogenic properties of DAT-1 (Carvelli et al., 2008). Here we investigated the effects caused by reduced expression of the *unc-64* gene in the dopaminergic neurons on DAT-1 function and DA-mediated behaviors. Because the *unc-64* knockout animals die shortly after embryogenesis (van Swinderen et al., 2001), we created transgenic animals expressing heritable and cell-specific knockdown of the *unc-64* gene in the dopaminergic neurons (*pdat-1::unc-64 sas*). We found that the *pdat-1::unc-64 sas* animals show a slight but statistically significant reduction in body bends when tested in plates without bacteria but they swim normally. However, when treated with Amph, the percentage of animals exhibiting SWIP was significantly reduced with respect to wild type animals. These behavioral results were supported by *in vitro* data showing that cultured neurons isolated from *pdat-1::unc-64 sas* animals exhibit reduced DA uptake and reduced DA release induced by Amph. Taken together these data suggest that Syntaxin-1A is required to mediate the physiological and behavioral effects caused by Amph in *C. elegans*.

## Materials and Methods

### Worms Husbandry and Strains

All *C. elegans* strains were cultivated under standard conditions, in non-crowded conditions, at 20°C on NGM plates seeded with the OP50 or NA22 *Escherichia coli* strains (Brenner, 1974), except for worms that were grown on *E. coli* strain HB101. The N2 wild type (Bristol variety), RM2702 *dat-1(ok157) III*, CB1112 *cat-2(e1112) II*, OH7547 *otIs199* [*pcat-2::GFP*; *prgef-1::dsRed*; *rol-6 (su1006)*], and CB246 *unc-64(e246) III* strains were provided by the Caenorhabditis Genetics Center (CGC, University of Minnesota, United States). BY200 *vtIs1* [*pdat-1::GFP*; *rol-6(su1006)*] V strain was obtained from Dr. Randy Blakely at the Brain Institute, Florida Atlantic University. To knockdown GFP encoding gene (*gfp*) in the dopaminergic neurons under the promoter of *cat-2* (*pcat-2*), the following transgene was used: *gbEx525* [GBF312 *pcat-2::gfp RNAi sas*; *podr-1::RFP*]; whereas, under the promoter of *dat-1* (*pdat-1*) we used: *gbEx572* [GBF326 *pdat-1::gfp RNAi sas*; pJM371 *pelt-2::NLS::RFP*]. To knockdown the *dat-1* gene under *pdat-1*, the following transgenes were used: *gbEx584* [GBF334 *pdat-1::dat-1 RNAi sas*; *podr-1::RFP*; EM282 *pcat-2::GFP*] and *gbEx624* [GBF334 *pdat-1::dat-1 RNAi sas*; *podr-1::RFP*; GBF325 *pdat-1::GFP*]. To knockdown the *cat-2* gene: *gbEx574* [GBF327 *pdat-1::cat-2 RNAi sas*; *podr-1::RFP*]. To knockdown the *kal-1* gene: *gbEx599* [GBF339 *pdat-1::kal-1 RNAi sas*; *podr-1::RFP*; GBF325 *pdat-1::GFP*]. To knockdown the *unc-64* gene: *gbEx585* [GBF335 *pdat-1::unc-64 RNAi sas*; *podr-1::RFP*; EM282 *pcat-2::GFP*] and *gbEx613* [GBF335 *pdat-1::unc-64 RNAi sas*; *podr-1::RFP*; GBF325 *pdat-1::GFP*].

### Construction of Transgenes for Neuron-Specific Knockdown

The construction of transgenes for neuron-specific knockdown was made by PCR fusion as previously described (Esposito et al., 2007). Genomic sequences, corresponding to the target gene and to promoters were amplified separately from *C. elegans* genomic DNA, unless otherwise noted. The promoter regions of *cat-2* or *dat-1* genes were chosen based on the cis-regulatory modules (CRM) necessary to drive expression specifically in dopaminergic neurons (Flames and Hobert, 2009; Illiano et al., 2017). The promoter specific expression in dopaminergic neurons was experimentally controlled by fusing them to *gfp* and by confirming that both were expressed in all dopaminergic neurons and only in them (data not shown). For the *cat-2* promoter a 600 bp fragment, upstream of the ATG, was amplified using the following primers: Pf *cat-2*: ataataaaactgcgtggcgtg; Pr *cat-2*: ctcttccaatttttcaagggg. The nested primer used for the second step of fusion was: Pf^∗^2 *cat-2*: cgtgttgttaagaacgtgcttgatcg. For the *dat-1* promoter a 795 bp fragment, upstream of the ATG, was amplified using the following primers: Pf *dat-1*: aaagtctttctgcccacacaa; Pr *dat-1*: agtaaaccgtagcgggatcag. The nested primer used for the second step was: Pf^∗^ *dat-1*: cgacctcatacactttctctcg. To amplify the *C. elegans* target genes to be silenced, we amplified the same exon rich regions that have been used for RNAi by feeding experiments (Kamath et al., 2003). For *gfp* expression and knockdown the fragment to be fused were amplified from A. Fire (Stanford University, United States) plasmids pPD95.75 and L4417, respectively. Primers used to fuse the *gfp* fragment to *dat-1* promoter for expression were: Tf *pdat-1::gfp sas*: ctgatcccgctacggtttactTCACTATAGGGAGACCGGCA; Tr *pdat-1::gfp sas*: ctgatcccgctacggtttactTCACTATAGGGCGAATTGGG. Primers used to fuse the *gfp* fragment to *cat-2* promoter for expression were: Tf *pcat-2::gfp sas*: ccccttgaaaaattggaagagTCACTATAGGGAGACCGGCA; Tr *pcat-2::gfp sas*: ccccttgaaaaattggaagagTCACTATAGGGCGAATTGGG. Primers used to amplify the *gfp* region (890 bp) for knockdown were: Tfa *gfp sas*: gttgtaaaacgacggccagt; Tfs *gfp sas*: GGCCGATTCATTAATGCAG; Tf^∗^ *gfp sas*: tcactataGGGAGACCGGCA; Tr^∗^ *gfp sas*: tcactatagggcgaattggg. Primers used to amplify the *cat-2* region (1092 bp) for knockdown were: Tfa *cat-2 sas*: caagctcttgtgatccgtga; Tfs *cat-2 sas*: acaatctgctgaacgccttt Tf^∗^ *cat-2 sas*: GAAATTCTCGATTTTCGCCA; Tr^∗^ *cat-1 sas*: CTTCTTTGCACAACCCGAAT. Primers used to fuse the *cat-2* fragment to *dat-1* promoter for knockdown were: Tf *pdat-1::cat-2 sas*: ctgatcccgctacggtttactGAAATTCTCGATTTTCGCCA; Tr *pdat-1::cat-2 sas*: ctgatcccgctacggtttactCTTCTTTGCACAACCCGAAT. Primers used to amplify the *dat-1* region (1190 bp) for knockdown were: Tfs *dat-1 sas*: TTCGAACCTGATCTCAACCC; Tfa *dat-1 sas*: TGCAGTTGGTGCCTACAGG; Tf^∗^ *dat-1 sas*: AAGCAAATGCACCGAACTCT; Tr^∗^*dat-1 sas*: AGCTCCAGCAAAACTTCCAA. Primers used to fuse the *dat-1* fragment to *dat-1* promoter for knockdown were: Tf *pdat-1::dat-1 sas*: ctgatcccgctacggtttactAAGCAAATGCACCGAACTCT; Tr *pdat-1::dat-1 sas*: ctgatcccgctacggtttactAGCTCCAGCAAAACTTCCAA. Primers used to amplify the *unc-64* region (1999 bp) for knockdown were: Tfa1 *unc-64 sas*: cttttcgtgtcgagacctgtc; Tfs *unc-64 sas*: AATGCCAGGAATATACTGAATGAG; Tr^∗^ *unc-64* (1) *sas*: CTCAATTCGATCAACCATCTCTC; Tf^∗^ *unc-64* (1) *sas*: AGAGATTCGTGGAAGTGTGGATA. Primers used to fuse *unc-64* fragments to *dat-1* promoter for knockdown were: Tf *pdat-1::unc-64 sas*: ctgatcccgctacggtttactAGAGATTCGTGGAAGTGTGGATA; Tr *pdat-1::unc-64 sas*: ctgatcccgctacggtttactCTCAATTCGATCAACCATCTCTC. All the Tf and Tr primers had at their 5′-end 20/21 additional nucleotides complementary to 3′ end of the promoter used to drive the knockdown. A mixture of sense and anti-sense PCR fusion product at the concentration of 50 ng/μL was microinjected together with co-transformation markers into the gonad of animals using standard microinjection technique (Mello et al., 1991). The following co-transformation markers were injected at the concentration of 30 ng/μL: p*odr-1::RFP*, expressed in the AWB and AWC neurons, a kind gift from C. Bargmann, Rockefeller University, United States; pJM371 p*elt-2::NLS::RFP*, expressed in intestinal cell nuclei, a kind gift from J. D. McGhee, University of Calgary, United States; EM#282 *pcat-2::GFP* expressed in dopaminergic neurons, a kind gift from S. Emmons, Albert Einstein College of Medicine, New York, United States. To test the silencing of *gfp* under the control of *dat-1* or *cat-2* promoters, the two PCR constructs were injected in two different integrated transgenic strains, in which the *gfp* is expressed in dopaminergic neurons with a “complementary” approach, i.e., *pdat-1::gfp (RNAi sas)* silencing construct was injected in *otIs199* [p*cat-2::GFP*] transgenic strain and vice versa, *pcat-2::gfp (RNAi sas)* silencing construct was injected in BY200 (*vtIs1* [p*dat-1::GFP*; *rol-6(su1006)*]) transgenic strain to avoid any disturbance of the same promoter on gene knockdown and on gene expression. To follow dopaminergic neurons in cultures some of the transgenic lines (i.e., *gbEx624, gbEx613*, and *gbEx599*) were obtained adding to the injection mix also GBF325 *pdat-1::GFP* fusion construct at 1 ng/μL. At least two lines for each genotype were analyzed in all cases and data pooled together.

### Microscopy and Imaging

Animals were mounted and anesthetized with 0.01% tetramisole hydrochloride (Sigma-Aldrich, St. Louis, MO, United States) on 4% agar pads. The analysis of GFP expression and *gfp* knockdown in dopaminergic neurons was performed using Zeiss Axioskop microscopes (Carl Zeiss, Oberkochen, Germany).

### Basal Slowing Response Assay

Basal slowing response assay was performed as previously described (Chase et al., 2004). Young adults, 18–22 h post-L4 stage at 20°C, were assayed. The locomotion rate of young adult animals was quantified by counting the number of body bends completed in five consecutive 20-s intervals in the presence or in absence of HB101 bacteria. Data were collected for six animals per condition, for a total of 30 measurements per condition. The percent of slowing was calculated by dividing the difference between locomotion rates on and off food by the locomotion rate off food.

### Swimming-Induced Paralysis Assays

Animals were grown in agar plates seeded with NA22 bacteria and SWIP assays were performed as previously described (Carvelli et al., 2010). Briefly, in each SWIP trial, 8–16 age-synchronized larva-4 animals were placed in 40 μl of vehicle (200 mM sucrose) with or without 0.5 mM Amph (NIDA, Research Triangle Institute) in a single well of a Pyrex spot plate (Thermo Fisher Scientific, Waltham, MA, United States). Paralyzed animals were counted after 10 min using an inverted microscope (Carl Zeiss, Inc., Thornwood, NY, United States). The number of paralyzed animals was reported as a percentage of the total number of animals observed in each test ± standard error.

### *Caenorhabditis elegans* Primary Cultures, [^3^H]DA Uptake and Release Experiments

We prepared embryonal cultures from animals grown on NA22 bacteria, as previously described (Carvelli et al., 2004). Briefly, 2-day-old embryonic cells (10^6^ cells/well) were pre-loaded with 5 nM [^3^H]DA (NEN) for 30 min at room temperature. Cells were washed five times with bath solution containing 145 mM NaCl or NMDG^+^, 5 mM KCl, 1 mM CaCl_2_, 5 mM MgCl_2_, 10 mM HEPES, and 20 mM D-glucose (pH 7.2 and 350 osmolarity) and 100 μM Amph or bath solution were then applied for 1 min to induce DA release. Samples were collected and counted for radioactivity. For uptake experiments 2 × 10^6^ cells were plated per well. Two days after seeded, cells were washed with bath solution and incubated with 50 nM [^3^H]DA for 5 min at room temperature. Uptake was terminated by washing the cells three times with ice-cold bath solution. Cells were quickly lysed using 1% SDS and samples were collected in vials to count radioactivity.

### Statistical Analysis

GraphPad Prism software (GraphPad Software, Inc., San Diego, CA, United States) software was used for statistical analyses. The statistical significance was determined using one-way ANOVA with Bonferroni post-test, Kruskal–Wallis and Student’s *t*-tests. The SWIP data passed the Shapiro–Wilk normality test (α = 0.05). Data are reported as averages of multiple observations ± Standard error.

## Results

### Cell-Specific RNAi Efficiently Silences Genes in Dopaminergic Neurons

**FIGURE 1:**
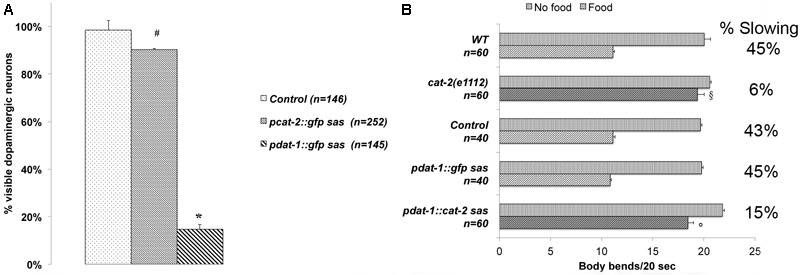
**(A)** *dat-1* promoter is more efficient in silencing GFP expression in the dopaminergic neurons than the *cat-2* promoter. Using a cell-specific RNAi technique (Esposito et al., 2007), the two promoters *pcat-2* and *pdat-1* were employed to silence the *gfp* gene specifically expressed in the dopaminergic neurons. Animals carry an integrated transgene for the expression of GFP only in dopaminergic neurons. The percentage of visible dopaminergic neurons expressing GFP over the number of neurons expected (eight per animal) is reported. *pdat-1* promoter was significantly more efficient (^∗^*p* < 0.001, control vs *pdat-1::gfp sas*) in silencing the GFP expression than *pcat-2* (^#^*p* > 0.01, control vs *pcat-2::gfp sas*). *n* is the number of animals observed. Statistical analysis was performed using one-way ANOVA with Bonferroni post-test. **(B)** *cat-2* silencing through the *dat-1* promoter is sufficient to mimic a *cat-2* dependent behavior. Similar to *cat-2(e1112)* null mutants (Sawin et al., 2000), *cat-2* silenced animals (*pdat-1::cat-2 sas*) do not slowdown in presence of food. On the other hand, wild type, controls (non-transgenic siblings) and GFP silenced (*pdat-1::gfp sas*) animals reduce their locomotion rate when encounter bacteria. The locomotion rate for 20 s off and on food is reported as percent of slowing (% Slowing) calculated by dividing the difference between locomotion rates on and off food by the locomotion rate off food. *n* represents the number of animals tested. Kruskal–Wallis non-parametric with Dunn’s multiple comparison post-test shows significant difference between wild type animals on food vs *cat-2(e1112*) on food (^§^ *p* < 0.001) and *pdat-1::gfp sas* on food vs *pdat-1::cat-2 sas* on food (°*p* < 0.001).

*unc-64* null mutants exhibit larval lethality, locomotion abnormalities, pharyngeal pumping and defecation defects (van Swinderen et al., 2001). Knockdown of *unc-64* using systemic RNAi causes growth and locomotion defects (Shephard et al., 2011). These defects do not allow using these deficient *unc-64* animals to perform behavioral assays and to explore the role of *unc-64* specifically in the dopaminergic neurons. To dissect the role played by *unc-64* specifically in dopaminergic neurons, in otherwise wild type animals, we created transgenic animals in which a dopaminergic specific promoter drives the expression of part of the gene, in the sense and antisense directions (RNAi sas) (Esposito et al., 2007; Gallotta et al., 2016). We initially tested the specificity and the efficiency of two dopaminergic-specific promoters, *pcat-2* and *pdat-1* (Flames and Hobert, 2009). The *cat-2* gene encodes the enzyme homolog to tyrosine hydroxylase, the rate-limiting enzyme required to produce DA. The *dat-1* gene encodes the DA transporter homolog to DAT, required to regulate synaptic DA signaling by controlling extracellular DA levels (Jayanthi et al., 1998). In *C. elegans*, these genes have been described to be specifically expressed only in the dopaminergic neurons (Flames and Hobert, 2009; Illiano et al., 2017). After confirming the dopaminergic-specific expression of *pcat-2* and *pdat-1* using a reporter gene (data not shown), we tested their efficiency in silencing the GFP in transgenic lines where GFP is constitutively expressed in all dopaminergic neurons (**Figure 1A**). The *pdat-1* promoter was largely more efficient in silencing the *gfp* (strain *pdat-1::gfp sas*), with only 15% of all dopaminergic neurons detectable by fluorescence microscopy (*p* < 0.001 vs control), while when using *pcat-2* (strain *pcat-2::gfp sas*), 90% GFP-positive neurons were still detectable, a percentage very similar to controls (98%). These results showed that the transgene driven neuron-specific silencing technique (RNAi sas) can be successfully applied to the dopaminergic neurons, that *pdat-1* is the appropriate promoter for this approach and we therefore used only this promoter in all the experiments. We then tested the efficiency of the *dat-1* promoter in silencing a gene known to play a function in the dopaminergic neurons. Thus, we evaluated the ability of *pdat-1* to knockdown *cat-2* gene by performing BSR experiments. This behavioral assay tests the ability of wild type animals to slow down their rate of locomotion when they encounter a bacterial lawn. This behavior is mediated by DA; in fact, *cat-2* null mutants do not exhibit BSR (Sawin et al., 2000). We used the BSR assay to evaluate the efficiency of the *cat-2* gene knockdown in dopaminergic neurons using the RNAi sas technique (**Figure 1B**). We observed that, similar to *cat-2(e1112)* null mutants, the transgenic animals silenced for *cat-2* (*pdat-1::cat-2 sas*) exhibited a defective BSR with respect to control animals (*pdat-1::gfp sas*). By representing BSR as the ratio between the locomotion rate off food and on food (% of Slowing in **Figure 1B**) we found that, similar to the *cat-2(e1112)* null mutants, the *pdat-1::cat-2 sas* animals exhibited a reduction in BSR, 6 and 15%, respectively. On the other hand, both the *pdat-1::gfp sas* animals (controls) and the wild type animals exhibited normal BSR values (45%). These results demonstrate that the RNAi sas technique is efficient, gene specific, and allows altering a DA-mediated behavior.

### Dopamine Neuron-Specific Silencing of *unc-64* Causes DA-Dependent Behaviors

**FIGURE 2:**
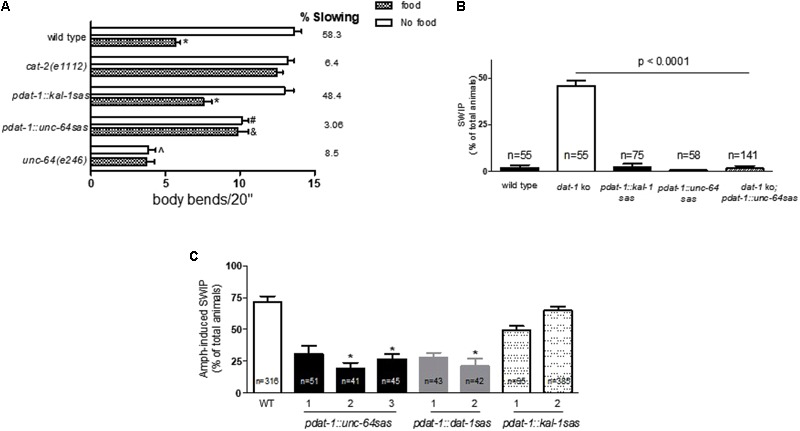
Dopamine neuron-selective *unc-64* knockdown alters DA-dependent behaviors. **(A)** Similarly to *cat-2(e1112)* knockouts (6.4%), the *pdat-1::unc-64 sas* lines exhibit highly reduced BSR (3%) with respect to wild type and *pdat-1::kal-1 sas* animals, 58 and 48%, respectively (^∗^*p* < 0.0001). In presence of food, the *pdat-1::unc-64 sas* mutants show a significant 33% increase (^&^*p* < 0.001) in body bends with respect to the control (*pdat-1::kal-1 sas*). The *pdat-1::unc-64 sas* mutants exhibit a slight but significant 21% reduction in body bends in absence of food with respect to *pdat-1::kal-1 sas* animals (^#^*p* < 0.001). Under the same conditions, the *unc-64(e246)* mutants exhibit a 71% reduction in body bends with respect to wild type animals (ˆ*p* < 0.0001). Animals were assayed as in **Figure 1B**. At least 18 animals were tested per each sample. **(B)** *unc-64* silencing in the dopaminergic neurons recovers the *dat-1* knockout phenotype. *dat-1 ko* animals exhibit SWIP when forced to swim for 10 min (McDonald et al., 2007). This phenotype was recovered in the *dat-1 ko;pdat-1::unc-64 sas* double mutants (Kruskal–Wallis non-parametric test). Wild type, *pdat-1;kal-1 sas* (controls) and *pdat-1::unc-64* animals do not exhibit SWIP. **(C)** Similarly to *dat-1* null mutants (Carvelli et al., 2010) and the two *dat-1* silenced strains (*pdat-1::dat-1 sas*), animals that are silenced for *unc-64* specifically in the dopaminergic neurons using the *dat-1* promoter (*pdat-1::unc-64 sas*) exhibit reduced Amph-induced swimming-induced paralysis (SWIP). Two lines of *kal-1* silenced animals (*pdat-1::kal-1 sas*), used as negative control, exhibit AMPH-induced SWIP values that are comparable to those observed in wild type animals. The three black bars for *pdat-1::unc-64 sas*, two gray bars for *pdat-1::dat-1 sas* and two dotted bars for *pdat-1::kal-1 sas* represent independent lines. In **(A–C)**, statistical analysis was performed using one-way ANOVA followed by both Bonferroni and Kruskal–Wallis non-parametric post-tests. *n* represents the number of animals tested per each strain.

The results shown in **Figure 1B** encouraged us to apply the RNAi sas technique to specifically knockdown *unc-64* in the dopaminergic neurons. Therefore using the *dat-1* promoter, we created the *pdat-1::unc-64 sas* mutants. Contrary to what observed in *unc-64* null or hypomorphic mutants or after systemic RNAi (van Swinderen et al., 2001; Shephard et al., 2011), the *pdat-1::unc-64 sas* animals were viable and did not present obvious developmental defects. To assess whether *unc-64* was knocked down in the dopaminergic neurons, we tested these mutants for BSR. As mentioned above, the BSR phenotype depends on DA and specifically extracellular DA released by the dopaminergic neurons. Since, UNC-64 is an essential factor for vesicular fusion and neurotransmitter release, we hypothesized that the lack of function of UNC-64 would prevent DA release and consequently would cause defective BSR. Our results show that like the null mutants *cat-2(e1112)*, which cannot synthesize DA, the *pdat-1::unc-64 sas* lines, which cannot release DA via vesicle fusion, failed to show BSR, 6.4 and 3%, respectively (**Figure 2A**). For these experiments we created, as negative control, transgenic animals that were knocked down for the *kal-1* gene using *pdat-1* (*pdat-1::kal-1 sas*). We reasoned that *kal-1* silencing represents the proper control because, while *gfp* is exogenously injected in worms to create transgenic animals, *kal-1* is natively expressed in most *C. elegans* neurons (Rugarli et al., 2002). As expected, both the wild type and *pdat-1::kal-1 sas* animals exhibited BSR when encountering food (58.3 and 48.4%, respectively; **Figure 2A**). In absence of food, we observed a slight but statistically significant 23% reduction (^#^*p* < 0.001) of body bends in the *pdat-1::unc-64 sas* animals with respect to controls (*pdat-1::kal-1 sas*). These data suggest that the *pdat-1::unc-64 sas* animals have a modest reduction in locomotion. To better understand the extent of this reduction, we tested the hypomorphic *unc-64(e246)* mutant. Although non-lethal, the *unc-64(e246)* mutation produces a point mutation in the *unc-64* gene (Ogawa et al., 1998) which causes severe locomotion defects. In fact, in our BSR experiments the *unc-64(e246)* animals besides lacking the BSR phenotype, also showed a strong reduction of body bends (4 ± 0.4) in absence of food with respect to wild type animals (13.6 ± 0.5; ˆ*p* < 0.0001). Since the number of body bends in absence of food of the *pdat-1::unc-64 sas* animals was significantly higher (10 ± 0.4) then those observed in *unc-64(e246)* mutants, this result might suggest that in the *pdat-1::unc-64 sas* mutants the *unc-64* silencing occurs in a restricted number of cells, most likely the dopaminergic neurons, rather than malfunctioning in every cells, as in *unc-64(e246)* animals. On the other hand, the number of body bends of the *pdat-1::unc-64 sas* tested on food was significantly higher than those measured in the *pdat-1::kal-1 sas* animals (^&^*p* < 0.001, Kruskal–Wallis test). This result shows that *pdat-1::unc-64 sas* mutants are impaired in the BSR behavior compared to the control animals (*pdat-1::kal-1 sas* and wild type).

To further investigate the lack of function of UNC-64 in the dopaminergic neurons in the *pdat-1::unc-64 sas* animals, we utilized another DA-dependent behavior: SWIP. Previously, we showed that when immersed in liquid solutions, wild type worms swim vigorously for hours; however, *dat-1* null mutants exhibit SWIP, i.e., animals sink to the bottom of the well and do not move (McDonald et al., 2007). Since we showed that SWIP is in part caused by an increase of extracellular DA due to lack of function of DAT-1 (McDonald et al., 2007; Carvelli et al., 2008, 2010), we hypothesized that SWIP observed in *dat-1* null mutants can be recovered by knocking down *unc-64* in the dopaminergic neurons. In this case, even if DAT-1 cannot reuptake DA (*dat-1 ko*), the animals would not show SWIP because DA cannot be released (*pdat-1::unc-64 sas*). Thus, using the *pdat-1*, we knocked down *unc-64* in the *dat-1* null mutants and create the *dat-1 ko;pdat-1::unc-64 sas* double mutants. We found that the double mutants did not exhibited SWIP (*p* < 0.001 vs *dat-1 ko*, **Figure 2B**), confirming therefore that the RNAi sas technique we used, causes genetic ablation of *unc-64* in the dopaminergic neurons and, as a consequence, it prevents DA release. No SWIP was observed in control animals (wild type and *pdat-1::kal-1 sas*) or *pdat-1::unc-64 sas* mutants (**Figure 2B**). Taken together, these results suggest that the *pdat-1::unc-64 sas* mutants have reduced ability to release DA most likely because of the reduced expression of *unc-64* in the dopaminergic neurons.

### Dopamine Neuron-Specific Silencing of *unc-64* Reduces Amph-Induced Behaviors

Previously, we demonstrated that Amph, a drug that increases extracellular DA (Di Chiara and Imperato, 1988), causes SWIP in *C. elegans* (Carvelli et al., 2010). Here we investigated whether reduced expression of *unc-64* in the dopaminergic neurons (*pdat-1::unc-64 sas*) would alter Amph-induced SWIP. We found that three independent transgenic lines of *pdat-1::unc-64 sas* animals treated with Amph exhibited significantly reduced SWIP with respect to wild type animals (black bars in **Figure 2C**; ^∗^*p* < 0.0001). We also measured SWIP in transgenic animals that were silenced for the *dat-1* (*pdat-1::dat-1 sas*) or *kal-1* (*pdat-1::kal-1 sas*) genes, used as positive and negative controls, respectively. Interestingly, the reduction observed in the *pdat-1::unc-64 sas* animals was comparable to that observed in the two independent transgenic lines of *pdat-1::dat-1 sas* animals (gray bars in **Figure 2C**; ^∗^*p* < 0.0001). And, as expected, the two independent transgenic lines of *pdat-1::kal-1 sas* animals did not show significant change in Amph-induced SWIP with respect to wild type animals (dotted bars in **Figure 2C**). These results suggest that *unc-64* might play a role in the mechanism of action of Amph that ultimately generates SWIP.

### Dopaminergic-Specific Silencing of *unc-64* Reduces Amph-Induced DA Release and DA Uptake

**FIGURE 3:**
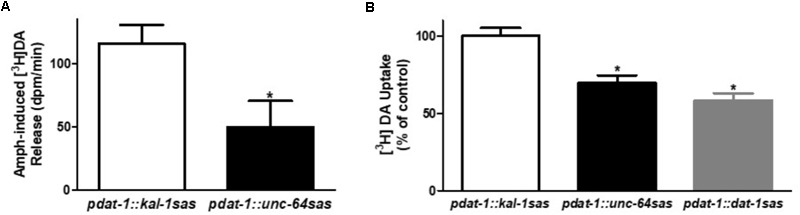
Dopamine neuron-selective *unc-64* knockdown decreases DA release and DA uptake. Cultured dopaminergic neurons isolated from transgenic animals were used to measure the Amph-induced DA release **(A)** and DA uptake **(B)**. **(A)** *unc-64* silenced cells (*pdat-1::unc-64 sas*) show a statistically significant reduction of [^3^H]DA release (^∗^*p* ≤ 0.0001) with respect to control cells (*pdat-1::kal-1 sas*). *t*-Test was used to performed statistical analysis. Data are presented as the difference of dpm (disintegration per minute) between samples treated with Amph and samples treated with control solution. **(B)** *pdat-1::unc-64 sas* cells show a statistically significant 30% reduction of [^3^H]DA uptake (^∗^*p* ≤ 0.001) with respect to control neurons (*pdat-1::kal-1 sas*). A similar reduction (40%) was observed in cells silenced for *dat-1* (*pdat-1::dat-1 sas*). Statistical analysis was performed using one-way ANOVA and Bonferroni post-test. Both data are average of values obtained from three independent experiments.

The strong reduction of Amph-induced SWIP measured in the *pdat-1::unc-64 sas* animals suggested that UNC-64, like its mammalian homolog Syntaxin-1A, is a protein required by Amph to induce DA efflux (Binda et al., 2008; Daberkow et al., 2013). To test this hypothesis we performed *in vitro* experiments using *C. elegans* primary cultures (Christensen et al., 2002; Carvelli et al., 2004). After preloading the embryonic cells with [^3^H]DA, cells were treated with Amph to induce release of [^3^H]DA. We found that cells derived from *pdat-1::unc-64 sas* animals had significant reduced [^3^H]DA release with respect to the control *pdat-1::kal-1 sas* cells (**Figure 3A**; ^∗^*p* < 0.05, *t*-test).

Previous studies showed that Syntaxin-1A interacts and regulates the activity of DAT (Binda et al., 2008; Carvelli et al., 2008; Cervinski et al., 2010). For example, Cervinski et al. (2010) showed that heterologous co-expression of Syntaxin-1A with rat DAT led to a reduction in DAT surface expression, which resulted in a reduction of DA uptake. We tested if this was also true in our native cultured cells by performing [^3^H]DA uptake experiments (**Figure 3B**). We found a significant reduction (31 ± 5%) in the uptake of the *pdat-1::unc-64 sas* cells with respect to the controls (*pdat-1::kal-1 sas* cells). Interestingly, this reduction was comparable to that obtained in *pdat-1::dat-1 sas* cells derived from animals in which *dat-1* gene was silenced using the RNAi sas technique (42 ± 5%). Because DA is accumulated inside the neurons by DAT, these results suggest that reduced expression of UNC-64 in the dopaminergic neurons causes a reduction of DAT activity or, as Cervinski et al. (2010) previously showed, a reduction of DAT expression on the cell membrane. Moreover, these results suggest that the reduced [^3^H]DA release observed in the *pdat-1::unc-64 sas* cells (**Figure 3A**) may result from less [^3^H]DA moving inside the neurons as seen in our uptake results (**Figure 3B**).

## Discussion

In the present study, we explored the role played by the Syntaxin-1A *C. elegans* homolog, *unc-64*, in the dopaminergic neurons. We used *C. elegans* because this model is amenable to genetic manipulations and conserves a high homology with the human dopaminergic-signaling pathway. To overcome the limitations presented by genetic null/hypomorphic mutants or systemic RNAi technique, we applied an alternative RNAi strategy, named RNAi sense and antisense (RNAi sas), to generate transgenic animals in which the function of *unc-64* gene was knocked down only in the dopaminergic neurons. This strategy, originally developed in our laboratory (Esposito et al., 2007), has been adopted by several groups to successfully silence various genes in almost all *C. elegans* tissues including neurons. In this study, we determined that the dopaminergic-specific promoter *pdat-1* is able to specifically and efficiently drive the knockdown of a reporter-gene, such as *gfp*, in the dopaminergic neurons only. The fact that *pdat-1* was more efficient than *pcat-2* in silencing the reporter gene may be due to a higher transcriptional activity of the promoter sequence we have chosen; hence a higher concentration of RNA silencing molecules was produced. Indeed, a clear difference in *gfp* intensity was visible when the same promoter was used to express the GFP as reporter gene (data not shown). The *dat-1* promoter sequence we chose contains three CRM or DA motifs, which are required and sufficient to drive expression in all dopaminergic neurons; on the other hand, the *cat-2* promoter we used had only one CRM motif (Flames and Hobert, 2009). Importantly, the *dat-1* promoter was very efficient in silencing genes that are endogenously expressed in the dopaminergic neurons, i.e., *cat-2* and *dat-1*, and therefore, *pdat-1* became the candidate promoter for silencing *unc-64* in these neurons.

Several studies have recognized the SNARE protein Syntaxin-1A as a key player of neurotransmitter release (Salaün et al., 2004) and one of the regulatory proteins of DAT. By binding to the DAT N-terminal domain (Lee et al., 2004), Syntaxin-1A modulates the release, transport, and ion channel activities of DAT (Binda et al., 2008; Carvelli et al., 2008; Cervinski et al., 2010). Thus, Syntaxin-1A may represent an important key element in the dopaminergic circuit that controls the amount of DA in the synaptic cleft. Using the RNAi sas technique, we created transgenic lines silenced for *unc-64* specifically in the dopaminergic neurons that are viable, able to grow and do not exhibit severe locomotor dysfunctions. This allowed us to overcome the limitations observed using classical *unc-64* RNAi, such as defects in growth and in locomotion (Shephard et al., 2011). Moreover, since the RNAi sas is a transgenic-based approach, the silencing constructs are heritable. This was a crucial requirement for the feasibility of both our *in vivo* and *in vitro* experiments (**Figures 2, 3**). Two distinct behavioral assays, BSR and SWIP, were used to test whether we effectively silenced the expression of *unc-64* in the dopaminergic neurons of *pdat-1::unc-64 sas* mutants. Both assays depend on the ability of the dopaminergic neurons to release DA, which ultimately makes the animals to slow down when they encounter food, BSR (Sawin et al., 2000), or to stop swimming if the DAT-1 function is ablated, SWIP (McDonald et al., 2007). The *pdat-1::unc-64 sas* mutants did not exhibit BSR, suggesting therefore that the lack of DA release is caused by the silencing of *unc-64* in the dopaminergic neurons (**Figure 2A**). Moreover, the SWIP behavior observed in the *dat-1 ko* mutants was recovered in the *dat-1 ko;pdat-1::unc-64 sas* double mutants, i.e., in animals that cannot release DA. Taken together, these results suggest that the RNAi sas strategy we used effectively silences *unc-64* in the dopaminergic neurons. We cannot exclude that the *pdat-1::unc-64 sas* mutants are deficient of *unc-64* in additional cells other than the dopaminergic neurons; however, we can speculate that the lack of obvious developmental defects seen in these animals makes this possibility quite unlikely. This hypothesis is also supported by our behavioral data (**Figure 2A**). In fact, while the hypomorphic *unc-64* mutants (*unc-64(e246)*) move very slowly in absence of food (four body bends/20 s), the *pdat-1::unc-64 sas* mutants move almost three times faster (10 body bends/20 s) than the *unc-64* hypomorphic mutants, and only slightly slower than the wild type animals (13 body bends/20 s).

Three *pdat-1::unc-64 sas* transgenic lines obtained with the RNAi sas technique (**Figure 2C**), exhibited a strong reduction in Amph-induced SWIP. Because the increase of extracellular DA is one of the causes generating Amph-induced SWIP (Carvelli et al., 2010; Safratowich et al., 2013, 2014), the reduction in SWIP seen in *pdat-1::unc-64 sas* animals treated with Amph could be due to a reduced amount of DA released in the synaptic cleft. We tested this hypothesis by performing *in vitro* assays, and we found that indeed the release of DA triggered by Amph was diminished in dopaminergic neurons of *pdat-1::unc-64 sas* mutants. Previous studies have shown that Amph evokes DA release through two separate mechanisms (Siciliano et al., 2014), one vesicle-independent and DAT-mediated (Sulzer et al., 1993; Jones et al., 1998) and one DAT independent and vesicle-mediated (Daberkow et al., 2013). Because UNC-64 is required to dock and fuse the vesicles at the cell membrane such that the neurotransmitter can be released, the reduced Amph-induced DA release measured in neurons silenced for *unc-64* could indicate that fewer vesicles empty their DA into the synaptic cleft. We also found that the reduced expression of *unc-64* in the dopaminergic neurons (*pdat-1::unc-64 sas*) diminished the DA uptake (**Figure 3B**). These results are in accordance with previous published data showing that Syntaxin-1A decreases DA uptake by reducing the amount of DAT on the cell membrane (Cervinski et al., 2010). If we assume that a reduction in the number of DAT proteins is responsible of the diminished DA uptake observed in the *pdat-1::unc-64 sas* neurons (**Figure 3B**), then we can speculate that less Amph is taken up inside these neurons since Amph, like DA, is a DAT substrate. This, in turn, would be the cause of the reduced DA release we measured in the *pdat-1::unc-64 sas* neurons (**Figure 3A**). In fact, with less Amph moved inside, we get less DA released out. Regardless of these speculations and interpretations, we may conclude here that in *C. elegans*, Syntaxin-1A besides controlling the basal release of DA also moderates the behavioral effects generated by Amph by reducing the amount of DA in the synaptic cleft.

## Author Contributions

AL carried out the molecular genetic manipulations, microscope analysis, and BSR assays. BDS performed SWIP assays, the [^3^H]DA release and uptake experiments. IG did the initial molecular genetic manipulations and initial characterization. GZ participated in the genetic manipulations. SRK performed the BSR assays. LC and EDS conceived, designed, coordinated the study and wrote the manuscript. All authors read and approved the final manuscript.

## Conflict of Interest Statement

The authors declare that the research was conducted in the absence of any commercial or financial relationships that could be construed as a potential conflict of interest.

## Abbreviations

*C. elegans*: *Caenorhabditis elegans*
BSR: basal slowing response
DAT: dopamine transporter
DA: dopamine
Amph: amphetamine
GFP: green fluorescent protein
sas: sense and antisense
RNAi: RNA interference
SWIP: swimming-induced paralysis.

## References

[B1] Arien H. Wiser O. Arkin I. T. Leonov H. Atlas D. (2003). Syntaxin 1A modulates the voltage-gated L-type calcium channel (Ca v 1.2) in a cooperative manner. 278 29231–29239. 10.1074/jbc.M301401200 12721298

[B2] Binda F. Dipace C. Bowton E. Robertson S. D. Lute B. J. Fog J. U. (2008). Syntaxin 1A interaction with the dopamine transporter promotes amphetamine-induced dopamine efflux. 74 1101–1108. 10.1124/mol.108.048447 18617632 PMC2728020

[B3] Brenner S. (1974). The genetics of *Caenorhabditis elegans*. 77 71–94. 10.1093/genetics/77.1.71 PMC1213120 4366476

[B4] Cartier E. Hamilton P. J. Belovich A. N. Shekar A. Campbell N. G. Saunders C. (2015). Rare autism-associated variants implicate syntaxin 1 (STX1 R26Q) phosphorylation and the dopamine transporter (hDAT R51W) in dopamine neurotransmission and behaviors. 2 135–146. 10.1016/j.ebiom.2015.01.007 25774383 PMC4353922

[B5] Carvelli L. Blakely R. D. DeFelice L. J. (2008). Dopamine transporter/syntaxin 1A interactions regulate transporter channel activity and dopaminergic synaptic transmission. 105 14192–14197. 10.1073/pnas.0802214105 18768815 PMC2528871

[B6] Carvelli L. Matthies D. S. Galli A. (2010). Molecular mechanisms of amphetamine actions in *Caenorhabditis elegans*. 78 151–156. 10.1124/mol.109.062703 20410438 PMC2912056

[B7] Carvelli L. McDonald P. W. Blakely R. D. DeFelice L. J. (2004). Dopamine transporters depolarize neurons by a channel mechanism. 101 16046–16051. 10.1073/pnas.0403299101 15520385 PMC528740

[B8] Cervinski M. A. Foster J. D. Vaughan R. A. (2010). Syntaxin 1A regulates dopamine transporter activity, phosphorylation and surface expression. 170 408–416. 10.1016/j.neuroscience.2010.07.025 20643191 PMC2933327

[B9] Chase D. L. Pepper J. S. Koelle M. R. (2004). Mechanism of extrasynaptic dopamine signaling in *Caenorhabditis elegans*. 7 1096–1103. 10.1038/nn1316 15378064

[B10] Christensen M. Estevez A. Yin X. Fox R. Morrison R. McDonnell M. (2002). A primary culture system for functional analysis of C. elegans neurons and muscle cells. 33 503–514. 10.1016/S0896-6273(02)00591-3 11856526

[B11] Condliffe S. B. Zhang H. Frizzell R. A. (2004). Syntaxin 1A regulates ENaC channel activity. 279 10085–10092. 10.1074/jbc.M313592200 14703519

[B12] Daberkow D. P. Brown H. D. Bunner K. D. Kraniotis S. A. Doellman M. A. Ragozzino M. E. (2013). Amphetamine paradoxically augments exocytotic dopamine release and phasic dopamine signals. 33 452–463. 10.1523/JNEUROSCI.2136-12.2013 23303926 PMC3711765

[B13] Deken S. L. Beckman M. L. Boos L. Quick M. W. (2000). Transport rates of GABA transporters: regulation by the N-terminal domain and syntaxin 1A. 3 998–1003. 10.1038/79939 11017172

[B14] Di Chiara G. Imperato A. (1988). Drugs abused by humans preferentially increase synaptic dopamine concentrations in the mesolimbic system of freely moving rats. 85 5274–5278. 10.1073/pnas.85.14.5274 2899326 PMC281732

[B15] Esposito G. Di Schiavi E. Bergamasco C. Bazzicalupo P. (2007). Efficient and cell specific knock-down of gene function in targeted *C. elegans* neurons. 395 170–176. 10.1016/j.gene.2007.03.002 17459615

[B16] Fan H.-P. Fan F.-J. Bao L. Pei G. (2006). SNAP-25/Syntaxin 1A complex functionally modulates neurotransmitter γ-Aminobutyric acid reuptake. 281 28174–28184. 10.1074/jbc.M601382200 16861228

[B17] Flames N. Hobert O. (2009). Gene regulatory logic of dopamine neuron differentiation. 458 885–889. 10.1038/nature07929 19287374 PMC2671564

[B18] Gallotta I. Mazzarella N. Donato A. Esposito A. Chaplin J. C. Castro S. (2016). Neuron-specific knock-down of SMN1 causes neuron degeneration and death through an apoptotic mechanism. 25 2564–2577. 10.1093/hmg/ddw119 27260405 PMC5181630

[B19] Geerlings A. Núñez E. López-Corcuera B. Aragón C. (2001). Calcium- and syntaxin 1-mediated trafficking of the neuronal glycine transporter GLYT2. 276 17584–17590. 10.1074/jbc.M010602200 11278707

[B20] Haase J. Killian A. M. Magnani F. Williams C. (2001). Regulation of the serotonin transporter by interacting proteins. 29 722–728. 10.1042/bst0290722 11709063

[B21] Horton N. Quick M. W. (2001). Syntaxin 1A up-regulates GABA transporter expression by subcellular redistribution. 18 39–44. 10.1080/09687680010029383 11396610

[B22] Illiano P. Lanzo A. Leo D. Paglione M. Zampi G. Gainetdinov R. R. (2017). A *Caenorhabditis elegans* model to study dopamine transporter deficiency syndrome. 45 207–214. 10.1111/ejn.13366 27519790

[B23] Jayanthi L. D. Apparsundaram S. Malone M. D. Ward E. Miller D. M. Eppler M. (1998). The *Caenorhabditis elegans* gene T23G5.5 encodes an antidepressant- and cocaine-sensitive dopamine transporter. 54 601–609. 9765501

[B24] Jones S. R. Gainetdinov R. R. Wightman R. M. Caron M. G. (1998). Mechanisms of amphetamine action revealed in mice lacking the dopamine transporter. 18 1979–1986. 10.1523/JNEUROSCI.18-06-01979.1998 PMC6792915 9482784

[B25] Kamath R. S. Fraser A. G. Dong Y. Poulin G. Durbin R. Gotta M. (2003). Systematic functional analysis of the *Caenorhabditis elegans* genome using RNAi. 421 231–237. 10.1038/nature01278 12529635

[B26] Khoshbouei H. Wang H. Lechleiter J. D. Javitch J. A. Galli A. (2003). Amphetamine-induced dopamine efflux: a voltage-sensitive and intracellular Na+-dependent mechanism. 278 12070–12077. 10.1074/jbc.M212815200 12556446

[B27] Lee K.-H. Kim M.-Y. Kim D.-H. Lee Y.-S. (2004). Syntaxin 1A and receptor for activated C kinase interact with the N-terminal region of human dopamine transporter. 29 1405–1409. 10.1023/B:NERE.0000026404.08779.43 15202772

[B28] Markota M. Sin J. Pantazopoulos H. Jonilionis R. Berretta S. (2014). Reduced dopamine transporter expression in the amygdala of subjects diagnosed with schizophrenia. 40 984–991. 10.1093/schbul/sbu084 24936023 PMC4133683

[B29] McDonald P. W. Hardie S. L. Jessen T. N. Carvelli L. Matthies D. S. Blakely R. D. (2007). Vigorous motor activity in *Caenorhabditis elegans* requires efficient clearance of dopamine mediated by synaptic localization of the dopamine Transporter DAT-1. 27 14216–14227. 10.1523/JNEUROSCI.2992-07.2007 18094261 PMC6673513

[B30] Mello C. C. Kramer J. M. Stinchcomb D. Ambros V. (1991). Efficient gene transfer in *C.elegans*: extrachromosomal maintenance and integration of transforming sequences. 10 3959–3970. 1935914 10.1002/j.1460-2075.1991.tb04966.x PMC453137

[B31] Miller G. W. Gainetdinov R. R. Levey A. I. Caron M. G. (1999). Dopamine transporters and neuronal injury. 20 424–429. 10.1016/S0165-6147(99)01379-6 10498956

[B32] Naren A. P. Quick M. W. Collawn J. F. Nelson D. J. Kirk K. L. (1998). Syntaxin 1A inhibits CFTR chloride channels by means of domain-specific protein-protein interactions. 95 10972–10977. 10.1073/pnas.95.18.10972 9724814 PMC28005

[B33] Ogawa H. Harada S. I. Sassa T. Yamamoto H. Hosono R. (1998). Functional properties of the unc-64 gene encoding a *Caenorhabditis elegans* syntaxin. 273 2192–2198. 10.1074/jbc.273.4.2192 9442061

[B34] Quick M. W. (2003). Regulating the conducting states of a mammalian serotonin transporter. 40 537–549. 10.1016/S0896-6273(03)00605-6 14642278

[B35] Quick M. W. (2006). The role of SNARE proteins in trafficking and function of neurotransmitter transporters. 175 181–196. 10.1007/3-540-29784-7_9 16722236

[B36] Rugarli E. I. Di Schiavi E. Hilliard M. A. Arbucci S. Ghezzi C. Facciolli A. (2002). The Kallmann syndrome gene homolog in *C. elegans* is involved in epidermal morphogenesis and neurite branching. 129 1283–1294. 11874923 10.1242/dev.129.5.1283

[B37] Safratowich B. D. Hossain M. Bianchi L. Carvelli L. (2014). Amphetamine potentiates the effects of -phenylethylamine through activation of an amine-gated chloride channel. 34 4686–4691. 10.1523/JNEUROSCI.3100-13.2014 24672014 PMC3965791

[B38] Safratowich B. D. Lor C. Bianchi L. Carvelli L. (2013). Amphetamine activates an amine-gated chloride channel to generate behavioral effects in *Caenorhabditis elegans*. 288 21630–21637. 10.1074/jbc.M113.484139 23775081 PMC3724622

[B39] Salaün C. James D. J. Greaves J. Chamberlain L. H. (2004). Plasma membrane targeting of exocytic SNARE proteins. 1693 81–89. 10.1016/j.bbamcr.2004.05.008 15313010

[B40] Sawin E. R. Ranganathan R. Horvitz H. R. (2000). *C. elegans* locomotory rate is modulated by the environment through a dopaminergic pathway and by experience through a serotonergic pathway. 26 619–631. 10.1016/S0896-6273(00)81199-X 10896158

[B41] Shephard F. Adenle A. A. Jacobson L. A. Szewczyk N. J. (2011). Identification and Functional clustering of genes regulating muscle protein degradation from amongst the known *C. elegans* muscle mutants. 6:e24686. 10.1371/journal.pone.0024686 21980350 PMC3181249

[B42] Siciliano C. A. Calipari E. S. Ferris M. J. Jones S. R. (2014). Biphasic mechanisms of amphetamine action at the dopamine terminal. 34 5575–5582. 10.1523/JNEUROSCI.4050-13.2014 24741047 PMC3988413

[B43] Sulzer D. Maidment N. T. Rayport S. (1993). Amphetamine and other weak bases act to promote reverse transport of dopamine in ventral midbrain neurons. 60 527–535. 10.1111/j.1471-4159.1993.tb03181.x 8419534

[B44] Sung U. Apparsundaram S. Galli A. Kahlig K. M. Savchenko V. Schroeter S. (2003). A regulated interaction of syntaxin 1A with the antidepressant-sensitive norepinephrine transporter establishes catecholamine clearance capacity. 23 1697–1709. 10.1523/JNEUROSCI.23-05-01697.2003 PMC6741950 12629174

[B45] Tsuk S. Michaelevski I. Bentley G. N. Joho R. H. Chikvashvili D. Lotan I. (2004). Kv2.1 channel activation and inactivation is influenced by physical interactions of both syntaxin 1A and the syntaxin 1A/Soluble N-Ethylmaleimide-Sensitive Factor-25 (t-SNARE) complex with the c terminus of the channel. 67 480–488. 10.1124/mol.104.005314 15525758

[B46] van Swinderen B. Metz L. B. Shebester L. D. Mendel J. E. Sternberg P. W. Crowder C. M. (2001). Goalpha regulates volatile anesthetic action in *Caenorhabditis elegans*. 158 643–655. 11404329 10.1093/genetics/158.2.643 PMC1461665

[B47] Wang D. Deken S. L. Whitworth T. L. Quick M. W. (2003). Syntaxin 1A Inhibits GABA flux, efflux, and exchange mediated by the rat brain GABA transporter GAT1. 64 905–913. 10.1124/mol.64.4.905 14500747

[B48] Wang G. J. Volkow N. D. Wigal T. Kollins S. H. Newcorn J. H. Telang F. (2013). Long-term stimulant treatment affects brain dopamine transporter level in patients with attention deficit hyperactive disorder. 8:e63023. 10.1371/journal.pone.0063023 23696790 PMC3655054

